# Effects of Shallow Needling for Chronic Primary Insomnia: Protocol for a Randomized Controlled Trial

**DOI:** 10.2196/76501

**Published:** 2025-11-11

**Authors:** Wanqing Lin, Chenlin Wang, Jiajia Ye, Yingling Ye, Min Tang, Qianqian Hu, Bin Chen

**Affiliations:** 1 Department of Rehabilitation Medicine and National Clinical Research Base of Traditional Chinese Medicine, The Affiliated People's Hospital of Fujian University of Traditional Chinese Medicine, No. 602, 817 Middle Road, Taijiang District, Fuzhou, 350004, China, 86 15980273832; 2 Department of Rehabilitation Assessments, Rehabilitation Hospital Affiliated to Fujian University of Traditional Chinese Medicine, Fuzhou, China

**Keywords:** shallow needling, chronic insomnia, acupuncture, study protocol, randomized controlled trial

## Abstract

**Background:**

Primary insomnia (PI), commonly identified by difficulties in initiating and maintaining sleep, negatively impacts both physical and mental health and increases the risk of occupational and vehicular accidents. Previous research has indicated that shallow needling, a form of acupuncture, may ameliorate the symptoms of PI. Nevertheless, the scientific evidence regarding its efficacy in enhancing sleep quality remains limited.

**Objective:**

This trial aims to assess the therapeutic efficacy of shallow needling in the treatment of chronic PI in adults.

**Methods:**

A single-center, prospective, assessor-blinded randomized controlled clinical trial retrospectively registered with the International Traditional Medicine Clinical Trial Registry (ITMCTR2024000409). With 2 parallel arms, the trial will be conducted at the Affiliated People’s Hospital of Fujian University of Traditional Chinese Medicine. A total of 124 participants with PI will be randomly divided into the control group and the treatment group in a ratio of 1:1 (n=62 for each group). The control group will receive 1 mg eszopiclone orally, once a day for 4 weeks. In addition to taking eszopiclone, the treatment group will receive shallow needling therapy once daily, 5 times a week, for 4 weeks. Data will be collected at 3 time slots—before treatment, after treatment, and 4 weeks after treatment—and will be analyzed using SPSS (version 23.0). The primary outcome measure is the Pittsburgh Sleep Quality Index. The secondary outcome measures include the Hamilton Anxiety Scale, Insomnia Severity Index, serum neurotransmitter detection (including dopamine, norepinephrine, and melatonin), sleep parameters, and magnetic resonance spectroscopy of the thalamus.

**Results:**

Participant recruitment for this study is currently in progress. The first participant was enrolled in August 2023, marking the official commencement of the trial. Following the completion of recruitment, data processing and statistical analysis will be initiated. The final results of this study are expected to be prepared and submitted for publication in January 2026.

**Conclusions:**

This study will evaluate the therapeutic effectiveness and safety of shallow needling in the treatment of chronic insomnia to provide the necessary scientific basis for the clinical application and promotion of shallow needling. The findings of this study may provide a scientific and standardized treatment protocol for shallow needling in adults with chronic insomnia.

## Introduction

Insomnia is one of the most common sleep disorders globally, characterized by difficulty in falling and staying asleep, early morning awakenings, and an inability to return to sleep [1]. It may occur secondary to psychiatric or organic disorders, the use of prescribed or illicit substances, alcohol consumption, or a combination of these factors. However, insomnia can also occur independently, known as primary insomnia (PI), which is driven by a psychophysiological hyperarousal process [2]. A recent large-scale study revealed that approximately 16.2% of adults worldwide experience insomnia [3], with PI accounting for an estimated 25% of the cases. Notably, many of these patients experience a chronic course of the disorder [4]. Chronic insomnia has been associated with a wide range of adverse health outcomes, including headaches, dizziness, hypertension, and various cardiovascular and cerebrovascular diseases, which collectively undermine physical health, reduce quality of life, and pose significant risks to public safety. These effects result in substantial harm to both individuals and society [5,6]. Therefore, chronic PI has become a critical public health issue in contemporary society.

Sedative drugs and cognitive behavioral therapy (CBT) are the primary interventions for insomnia [7]. Although sedative drugs have a fast onset of action, their adverse effects with long-term use are notable, including persistent sleep difficulties, performance issues, memory disorders, driving accidents, and withdrawal syndrome [8]. In contrast, CBT is widely recognized as the first-line treatment for chronic insomnia by recognized organizations, including the American College of Physicians [9], the American Academy of Sleep Medicine [10], and the European Sleep Research Society [7]. Despite its effectiveness for insomnia, CBT is not commonly used due to the intensive labor required and high costs, which may lead to low patient compliance [11]. Given these limitations, it is crucial to seek additional safe and effective nonpharmacological treatments to optimize existing therapies for chronic insomnia.

As an important component of traditional nondrug therapy, acupuncture has long been used for the treatment of insomnia in China [12] [1]. In recent years, a growing body of evidence-based research has reported the effectiveness and feasibility of acupuncture therapy in improving sleep quality [13-16]. However, in clinical practice, the discomfort and pain associated with traditional acupuncture often cause significant distress and fear among patients, which limits its widespread acceptance. Therefore, the development of noninvasive and painless acupuncture techniques has become a key focus of research in this field.

**Figure 1.**
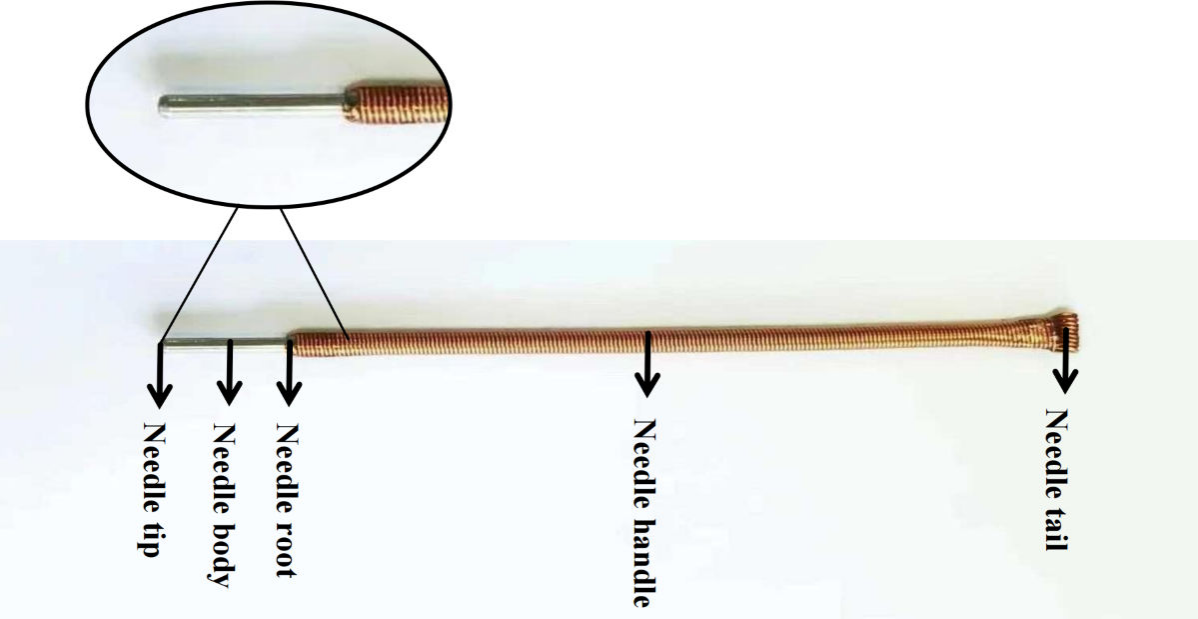
Structure of the shallow needle used in this study, illustrating the key components, including the needle tip, body, root, handle, and tail.

Shallow needling, a special type of acupuncture, is derived from the *hammer needle* in the ancient system of 9 needles. The nationally famous senior traditional Chinese medicine experts, Wu Binghuang and Liang Dongfu, have inherited and innovated this therapy, developing it into a characteristic acupuncture method of Fujian province [17]. In 2019, it was listed as part of the sixth batch of intangible cultural heritage projects in Fujian province and was selected as a characteristic traditional Chinese medicine technique by the National Administration of Traditional Chinese Medicine. Shallow needling is a noninvasive technique in which a rounded needle tip rests on the skin surface without penetrating it. Compared to conventional acupuncture needles, the needles used in shallow needling are designed with a thicker body, a longer handle to facilitate scraping with the fingernail, and a rounded tip that rests on the skin surface, avoiding penetration and minimizing discomfort (Figure 1). The technique involves applying a scraping motion with the fingernail on the needle handle at specific acupoints on the body surface to produce a needle sensation. This method promotes the flow of meridian Qi and blood, regulates the internal balance of the body, and treats various diseases.

As a noninvasive and painless alternative, shallow needling is an attractive option for patients seeking less discomfort during treatment. Although shallow needling shows promising potential, its clinical efficacy in treating insomnia has not been thoroughly explored. A recent systematic review and meta-analysis [18] demonstrated that compared to nonshallow needling treatments, shallow needling demonstrates greater efficacy in reducing Pittsburgh Sleep Quality Index (PSQI) scores and improving clinical efficacy rates. However, most studies have small sample sizes, unclear reporting, and potential bias risks, and there is still insufficient high-quality clinical evidence to support its effectiveness.

Furthermore, the specific mechanisms through which shallow needling ameliorates insomnia remain poorly understood, which has limited its broader clinical application. The current understanding of PI pathogenesis centers on hyperarousal as a core neurobiological pathology. Patients exhibit persistent whole-brain hyperexcitability, characterized by increased beta-band electroencephalographic activity. This abnormal cortical excitation interacts with the hypothalamic-pituitary-adrenal axis hyperactivity and autonomic nervous dysfunction, contributing to a vicious cycle of insomnia [19].

Existing evidence indicates that shallow needling restores autonomic balance [20] and mitigates the hypothalamic-pituitary-adrenal axis dysfunction, alleviating insomnia symptoms through correcting neuroendocrine abnormalities [21]. However, mechanistic research on the central nervous system effects of shallow needling remains inadequate, particularly regarding its impact on key brain regions regulating the sleep-wake cycle. Although preliminary studies have shown that shallow needling can regulate the functional connectivity of emotion-related brain circuits in patients with PI [22] and reduce the beta wave power in the frontal lobe and central regions of the brain [23], thereby creating neural conditions for sleep initiation, these findings still fail to systematically explain their core action targets.

The thalamus is a critical brain region involved in the regulation of sleep and wakefulness, and it plays a key role in sensory integration [24]. Neuroimaging studies have demonstrated that alterations in thalamic activity are associated with sleep disorders, including insomnia [25]. Recently, acupuncture has been shown to regulate the functional connections between the thalamus and the emotional network in patients with insomnia and improve the state of excessive arousal. Nevertheless, the underlying metabolic mechanisms remain unclear. Accumulating research has demonstrated that the occurrence of PI is closely linked to abnormal brain metabolites. Specifically, γ-aminobutyric acid (GABA) and glutamate/glutamine (Glx) maintain the balance between excitatory and inhibitory signals and are thought to be key players in sleep regulation. Notably, reduced GABA levels have been linked to hyperarousal in patients with insomnia, while abnormal Glx activity may contribute to disrupted sleep patterns [26]. Magnetic resonance spectroscopy (MRS) offers a noninvasive approach to quantify the concentrations of neurochemical substances within specific brain regions of living participants [27], providing a valuable tool for exploring the neurophysiological effects of acupuncture.

Therefore, we conducted a randomized controlled trial with a larger sample size and a rigorous design to generate high-quality clinical evidence regarding the efficacy of shallow needling for insomnia. We hypothesized that shallow needling acts as an effective external stimulus that modulates thalamic neurochemical activity through sensory nerve afferent pathways. To comprehensively evaluate its therapeutic effects, we incorporated peripheral serum neurotransmitter assays and objective polysomnographic sleep architecture measures, aiming to further elucidate the neural mechanism through which shallow needling ameliorates chronic PI.

## Methods

### Research Objectives

The purpose of this study is to evaluate the efficacy and safety of shallow needling in the treatment of chronic insomnia. The results of this study may provide a noninvasive and painless alternative therapy for patients with chronic insomnia.

### Hypotheses

We hypothesize the following:

1. Compared with the control group, the symptoms of insomnia and anxiety in the treatment group were significantly relieved after treatment according to the scale evaluation. At the same time, according to the objective sleep outcome test results, the sleep treatment of the treatment group was further proved to be improved.
2. Compared with the control group, the levels of serum neurotransmitters, central neurotransmitters, and metabolites in the treatment group were significantly improved, reflecting the better regulatory effect of shallow needling.

### Ethical Considerations

This study protocol has been approved by the ethics committee of the Affiliated People’s Hospital of Fujian University of Traditional Chinese Medicine (IRB 2023-014-02). All patients and participants will provide written informed consent before their participation in this study. To protect participants’ privacy and personal information in compliance with international ethical guidelines (eg, the World Medical Association Declaration of Helsinki), all identifiable data will undergo strict deidentification and anonymization processes immediately after data collection. This ensures that no individual participant can be traced or identified during data analysis, storage, or reporting stages. In recognition of participants’ time and travel costs incurred during the study, each participant will receive a one-time transportation subsidy of 450 CNY (approximately US $63). The subsidy is provided to offset travel expenses related to study visits and will be distributed upon completion of all required study procedures.

### Study Design

**Figure 2.**
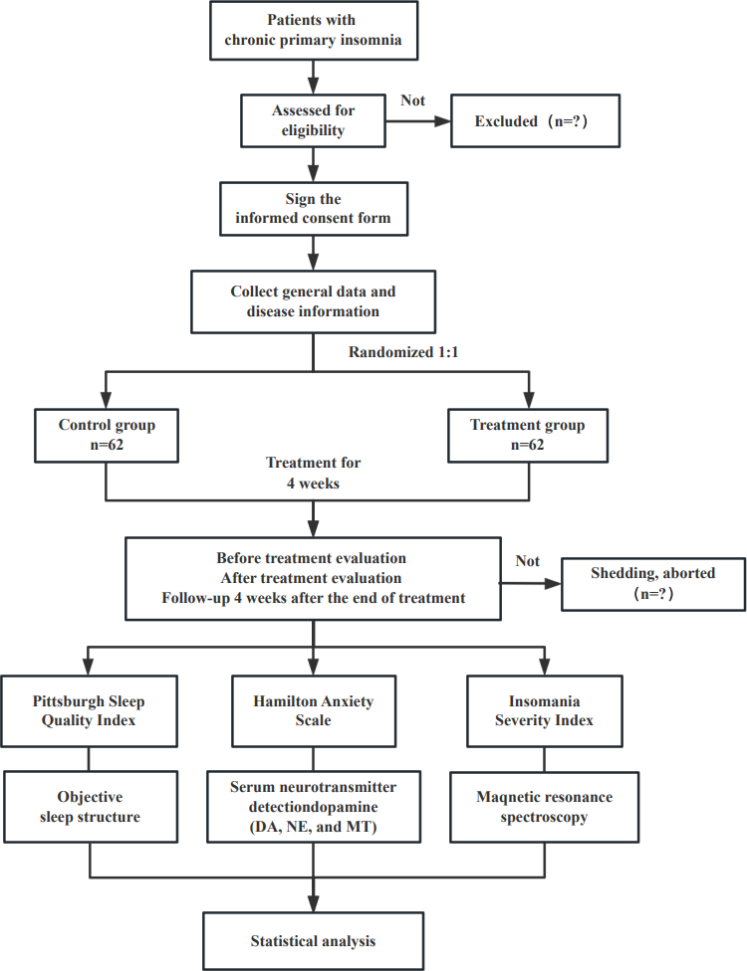
Flow diagram of the trial. DA: dopamine; MT: melatonin; NE: norepinephrine.

This study design is a prospective, randomized controlled trial with 2 parallel arms. The trial was retrospectively registered with the International Traditional Medicine Clinical Trial Registry under the number ITMCTR2024000409. The delay in registration occurred because the research team prioritized obtaining ethical approval and completing preliminary clinical preparations to ensure participant safety and study quality in the early stage; the registration process was initiated and completed immediately after the above preparatory work and subsequent administrative clearance were finalized. This protocol follows the guidelines of the Standard Protocol Items: Recommendations for Interventional Trials (SPIRIT) (Checklist 1). We will compare the differences between shallow needling and pharmacotherapy for chronic PI in adults. The study procedure and details are presented in Figures2  3.

**Figure 3.**
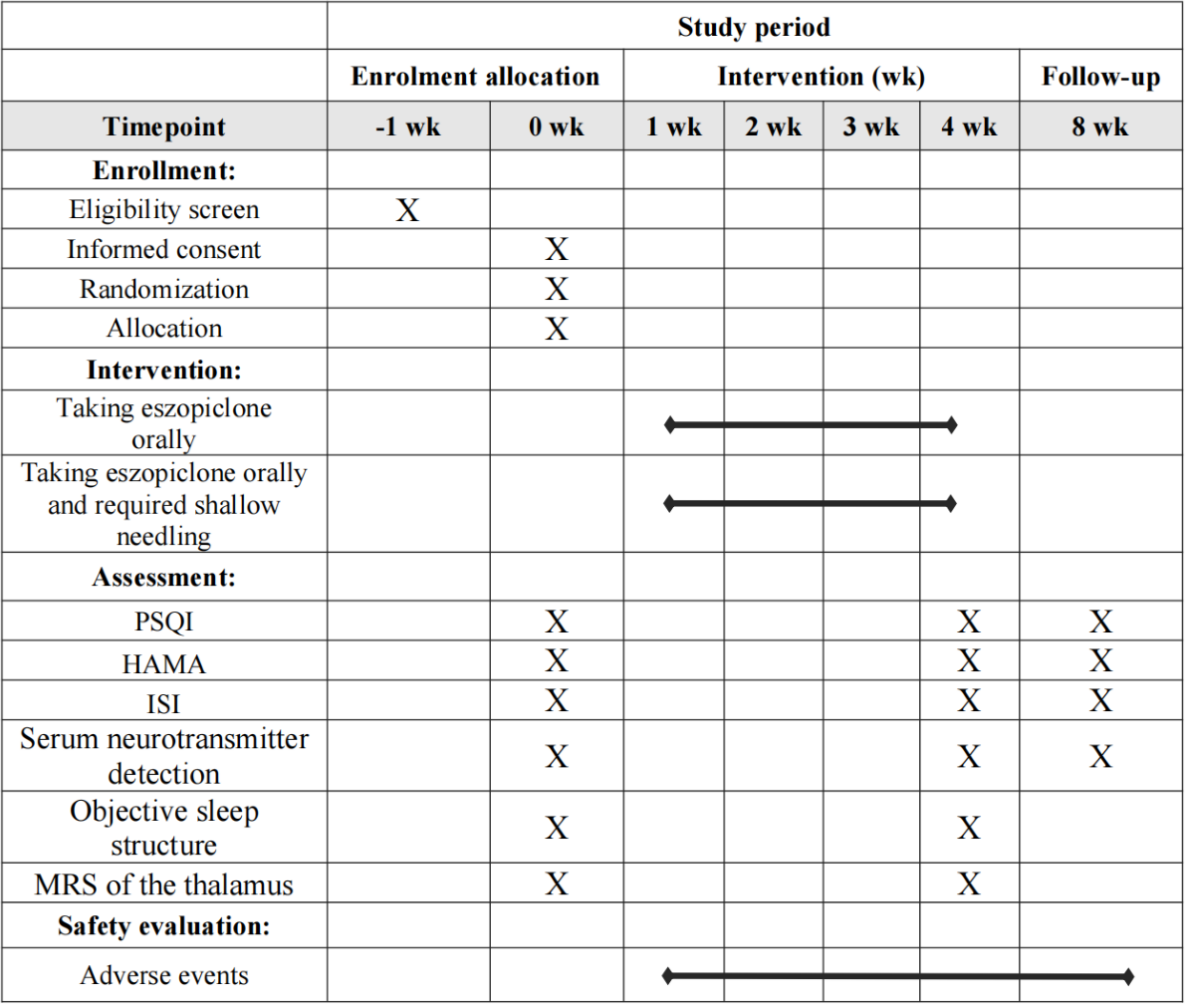
Study schedule of enrollment, interventions, and assessments. HAMA: Hamilton Anxiety Scale; ISI: Insomnia Severity Index; MRS: magnetic resonance spectroscopy; PSQI: Pittsburgh Sleep Quality Index.

### Participants

#### Sample Size

According to the relevant literature [28], the overall efficacy rate for the control group is reported to be 79.48%, whereas the treatment group is expected to have an efficacy rate of 98%. Assuming a significance level (α) of 0.05 and a power (1-β) of 0.9, the sample size was calculated using PASS 15.0 software. A minimum of 56 participants is initially required for each group. Considering a 10% dropout rate, we plan to recruit at least 62 participants per group. Therefore, a total of 124 participants are needed for this study.

#### Recruitment

We will recruit participants by posting advertisements on WeChat. Additionally, a series of educational lectures on shallow needling for chronic insomnia will be held at the Affiliated People’s Hospital of the Fujian University of Traditional Chinese Medicine to attract potential participants. All participants who meet the selection criteria will sign an informed consent form after understanding the purpose of the study and the program implementation process. Individuals’ personal information will be collected and securely stored in a separate cabinet to ensure confidentiality. The inclusion and exclusion criteria are detailed in Textbox 1.

Textbox 1. **Inclusion and exclusion criteria.**

**Inclusion criteria**

- Patients who meet the diagnostic criteria
- Patients with insomnia aged 18 to 65 years
- Pittsburgh Sleep Quality Index score range from 7 to 15
- The duration of the disease ≥3 months
- No communication and cognitive impairment
- Voluntary enrollment and can sign informed consent form

**Exclusion criteria**

- Secondary insomnia
- Unwillingness to receive shallow needling therapy
- Pregnant or lactating women, or those who intend to become pregnant
- Patients with heart, liver, kidney, lung, brain, and other serious organic diseases
- Participants in other studies that may influence the results of this study

#### Randomization and Blinding

##### Randomization

The randomization sequence will be computer-generated by an independent statistician using SPSS Statistics (version 23.0; IBM Corp), using a block randomization method with a 1:1 allocation ratio. Sequentially numbered, opaque, sealed envelopes will be used to implement allocation concealment. Each envelope will contain a card specifying the random number, sequence number, and group assignment. After obtaining written informed consent, eligible participants will be assigned to either the treatment group or the control group by opening the next consecutively numbered envelope, and it is ensured that the random allocation sequence remains unknown to the researchers before enrollment to prevent selection bias.

##### Blinding

Due to the specific nature of the shallow needling procedure, we could not blind the investigators and participants, but outcome assessors and statisticians would be unaware of the trial-group assignments.

### Interventions

#### The Control Group

Participants in the control group will receive oral eszopiclone (trade name: Itannin, manufactured by Chengdu Kanghong Pharmaceutical Group Co, Ltd, approval number: Sinopharm H20100074) at a dose of 1 mg before bedtime daily, for 4 weeks.

#### The Treatment Group

In addition to eszopiclone (same as the control group), participants will receive shallow needling therapy daily, 5 times a week, for 4 weeks.

**Figure 4.**
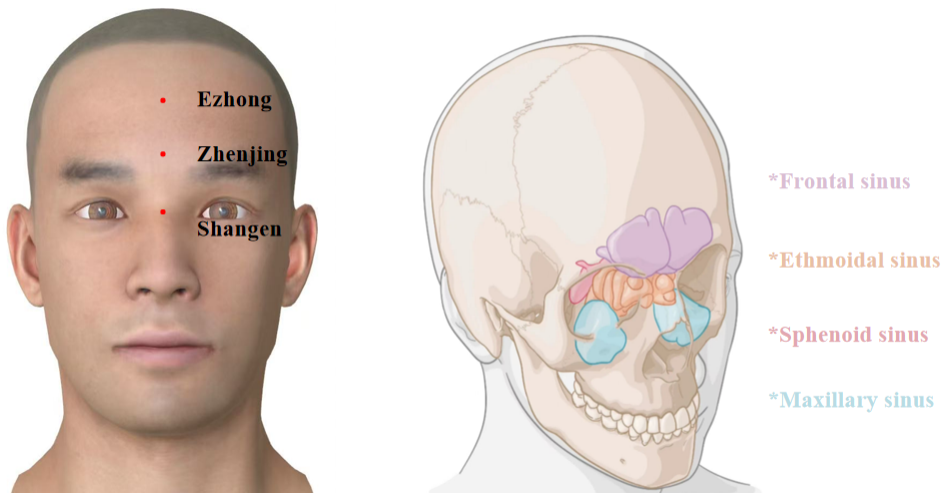
Anatomical locations of the acupoints.

The selected acupoints are Shangen (located at the intersection of the line connecting the inner canthi of both eyes and the midline of the nose), Zhenjing (located 0.3 cun above Yintang [GV29], with 1 cun being approximately the width of the patient’s thumb at the knuckle), and Ezhong (located at the midpoint of the line connecting the hairline and Yintang). The locations of the acupoints are shown in Figure 4.

Before treatment, the participants are told to lie in a supine position in a quiet environment. The practitioner wraps a small piece of disinfected cotton around the tip of the needle and places it on the acupoint, applying light pressure without penetrating the skin of the patient. The practitioner uses the pad of the thumb to stabilize the needle handle, ensuring that the tip is perpendicular to the surface of the acupoint. The practitioner uses the edge of the middle fingernail to continuously scrape upward and downward along the needle handle, creating gentle and uniform vibrations. Each cycle of 81 scraping motions is 1 dose of stimulation, with 3 doses per acupoint.

The shallow needling will be performed by 3 licensed acupuncturists with at least 5 years of clinical experience in acupuncture. They will receive comprehensive training on the study procedures and shallow needling techniques before participating in this study.

In order to ensure the accuracy and reliability of the results of this trial, the use of other disease-related treatments was prohibited during the trial.

#### Follow-Up

The aim of the follow-up procedure is to assess the long-term effects of shallow needling for chronic PI. Follow-up assessments will be conducted after the 4-week intervention program. No further interventions will be provided during this period. Participants will obtain the same outcome measures as before the intervention. Regular reminders will be provided to ensure adherence, and any adverse events will be recorded and monitored by the researchers. This follow-up procedure is essential for understanding the long-term effects of shallow needling.

### Outcome Measures

#### Primary Outcome Measures

PSQI [29] is a questionnaire used to assess an individual’s sleep quality, with good reliability and validity, indicated by a Cronbach α coefficient of 0.83 and a test-retest reliability of 0.85 [30]. It consists of 7 components: subjective sleep quality, sleep latency, sleep duration, habitual sleep efficiency, sleep disturbances, use of sleeping medication, and daytime dysfunction. The total score ranges from 0 to 21. A global PSQI score greater than 7 indicates the presence of sleep disorders, with higher scores reflecting poorer sleep quality. PSQI will be assessed before treatment, after treatment, and 4 weeks after treatment completion (follow-up).

#### Secondary Outcome Measures

##### Overview

Hamilton anxiety scale [31] consists of 14 items with a scale ranging from 0 to 4. Higher scores indicate more severe anxiety levels. This scale is usually used to measure the levels of anxiety. The evaluation time points are the same as those for the primary outcome indicator.

Insomnia Severity Index [32] is a self-assessment scale used to measure an individual’s experience of insomnia. It consists of 7 items, each rated on a scale from 0 to 4. Higher scores indicate greater severity of insomnia. The total score ranges from 0 to 28, categorized as follows: 0‐7 (no insomnia), 8‐14 (mild insomnia), 15‐21 (moderate insomnia), and 22‐28 (severe insomnia). Assessment time points align with the primary outcome measure.

##### Objective Sleep Structure

Overnight sleep monitoring will be conducted using a polysomnography device (model: Alice 6 LDxN/LDxS, purchased from Respironics). The parameters, including total sleep time, sleep latency, wake after sleep onset, sleep efficiency, and the percentage of total sleep time spent in rapid eye movement (REM) and non-REM sleep stages 1, 2, and 3/4 (with stages 3 and 4 combined as deep sleep, reported as REM%, N1%, N2%, and N3%) were selected for outcome measurement. Participants will undergo sleep monitoring from 7 PM to 7 AM on the day before treatment and the day after the completion of the treatment course in the hospital.

##### Serum Neurotransmitter Detection

Fasting blood samples (5 mL) will be collected from participants between 8 AM and 10 AM. Blood samples will be centrifuged at 3000 rpm to separate the serum and subsequently stored in a laboratory freezer at −80°C. The concentrations of dopamine, norepinephrine, and melatonin will be measured using enzyme-linked immunosorbent assay kits, according to the manufacturer’s instructions (Shanghai Enzyme-linked Biotechnology Co, Ltd). Measurements will be taken 1 day before treatment, 1 day after the completion of the treatment course, and at a 4-week follow-up.

##### Thalamic MRS

Scans will be performed with a 3.0T magnetic resonance scanner (Philips Ingenia CX 3.0T, Philips Healthcare). Participants will be required to keep their eyes closed, remain at rest, and stay awake during scanning. The bilateral thalamus will be selected as the region of interest, and all scans will be performed by the same experienced radiologist.

##### Imaging Parameters

The s2D_PRESS_144 sequence will be used for data acquisition, with a total scan duration of 5 minutes and 52 seconds. The field of view of the equipment will be anteroposterior, with a diameter of 230 mm. Right-left diameter will be 192 mm. The size of the acquisition voxel will be 10.0×10.1×15.0 mm. The echo time will be 144 milliseconds. The repetition time will be 2000 milliseconds. The acquisition matrix will be 23×19. The slice thickness will be 15 mm and the scan percentage will be 100%.

After the acquisition of MRS data, postprocessing will be executed using the Magnetic Resonance Spectro View software installed on the Intellispace Portal workstation. This postprocessing will mainly involve tasks such as noise reduction, frequency calibration, and baseline correction. Subsequently, the processed data will be analyzed to measure the levels of GABA, Glx, N-acetylaspartate, and choline-containing compounds in the thalamic region. The software will identify the characteristic peaks of these metabolites in the MRS spectra and calculate their relative concentrations based on the peak areas and predefined algorithms. MRS will be conducted before and 4 weeks after treatment to assess the changes in central neurotransmitter and thalamic metabolite levels.

### Adverse Event Monitoring

Adverse events in this study will be closely monitored, with all details recorded in the case report forms (CRFs), to ensure that patients receive timely and appropriate management. Adverse events, including discomfort at the shallow needling site and drug-related reactions including nausea, vomiting, and dizziness, will be documented. Any serious adverse events will be promptly reported to the ethics committee to ensure participant safety.

### Statistical Analysis

In this study, data will be analyzed with SPSS (version 23.0) software. Continuous variables will be reported as means and SDs, and categorical variables will be presented as frequencies or percentages. To compare continuous variables between the 2 groups, normality and homogeneity of variance will be initially evaluated. If these assumptions are satisfied, a 2-tailed *t* test will be used. Otherwise, a nonparametric rank-sum test will be used. Categorical variables will be analyzed using the chi-square test or Fisher exact test. *P* values of .05 or less will be considered statistically significant.

### Data Collection and Management

Data will be collected by dedicated staff at the study center, and both the electronic medical record and CRF will be used to collect the data. In principle, missing values are not allowed in the report form. Particularly, important measures (the primary safety measure) must be clearly reported. If a test result is zero or if measurement fails, corresponding symbols (eg, “0” for zero values and “NM” for “not measured”) will be used instead of leaving the field vacant. This ensures a clear distinction from missing values. If there are missing values, the original data should be searched first to determine whether the data are missing. If it is indeed missing, the data should be filled in. If the missing value is an important indicator related to safety, the participant should be informed to review it immediately to ensure their safety. If the missing values affect the outcome, the case will be excluded.

### Quality Control

All participants, including acupuncturists, evaluators, and statisticians, will be required to undergo training to ensure the quality of the studies. By formulating detailed and operable clinical trial specifications, the intervention will be based on careful adherence to the standard operating procedures.

All participants, including acupuncturists, evaluators, and statisticians, need to be trained to ensure the consistency of relevant data collection and the quality of research through the development of detailed and operational clinical trial protocols. The intervention will be based on careful adherence to standard operating procedures, and the acupuncturists will perform standardized shallow needling procedures according to the standard acupoint positioning. After the training, the assessment effects of the scale by outcome evaluators need to be checked for consistency, mainly including the same case being scored by independent examination by several outcome evaluators and the same case being scored multiple times by one evaluator to ensure consistency of the scores.

The investigator will fill in all cases truthfully and carefully according to the design requirements of the CRF. Study medical records and CRFs are original records and cannot be altered.

Laboratory data in clinical trials should be documented, and the original report or photocopy needs to be glued to the CRF.

In order to effectively improve the compliance of the participants, during the case screening and enrollment stage, it is necessary to maintain a patient and careful attitude; fully explain the purpose, process, and significance of the study to the participants; and ensure that the participants clearly understand the relevant content. At the same time, the participants will be clearly informed to strictly follow the established requirements to ensure the smooth progress of the study and the accuracy of the data.

## Results

Participant recruitment is currently underway. The first participant was enrolled in August 2023, marking the official commencement of the experiment. As of April 2025, a total of 120 participants have been enrolled in this clinical trial. The final results of the study are expected to be prepared and submitted for publication in January 2026.

## Discussion

This study is a randomized controlled clinical trial aimed at evaluating the clinical efficacy of shallow needling in the treatment of chronic PI and to further explore its underlying mechanism. The objectives include exploring a cost-effective, efficient, and safe standard therapy for chronic PI, as well as providing a scientific foundation for the clinical application and promotion of shallow needling therapy.

Through systematic data mining of extensive literature, we demonstrated that acupuncture points such as Shangen, Zhenjing, and Ezhong have been widely recognized for their efficacy in treating insomnia through shallow needling [33]. These acupoints correspond to the anatomical structures above the paranasal sinuses, where vibratory stimulation through shallow needling may induce resonance within the subcutaneous cavities. This resonant effect could further stimulate the Circle of Willis, thereby improving cerebral function and blood circulation [20]. A preliminary study indicates [34] that Professor Wu Binghuang frequently selected these acupoints in clinical practice, documenting significant improvement in over 80 patients with insomnia, many of whom experienced sleep onset during treatment. Furthermore, electroencephalography findings from the same study indicated that shallow needling can induce delta waves through regular and continuous vibratory stimulation. This mechanism shares similarities with the principle applied by hypnotists, who induce delta waves to treat insomnia specifically via the visual stimulation of a rhythmically swaying crystal ball. Our study will use these well-documented acupoints to improve therapeutic outcomes.

This trial will comprehensively assess the efficacy of shallow needling through both subjective and objective measures, including the PSQI, Hamilton Anxiety Scale, Insomnia Severity Index, and objective sleep monitoring via polysomnography. This multifaceted evaluation approach allows for a robust assessment of the treatment effects from various perspectives. Although previous studies have predominantly examined short-term outcomes, our study design incorporates a 4-week intervention period followed by a 4-week follow-up phase, enabling the investigation of both the immediate and sustained effects of shallow needling. This longitudinal design provides valuable insights into the therapeutic potential of the intervention. Additionally, to further elucidate the underlying neurophysiological mechanisms, we will analyze serum neurotransmitter levels and conduct thalamic MRS, exploring how shallow needling may modulate the neural pathways involved in sleep regulation. Moreover, this trial avoids the use of skin-penetrating interventions, ensuring participant safety and enhancing both participant compliance and the feasibility of the study.

Although our study comprehensively evaluates the efficacy of shallow needling for chronic PI, there are several limitations to consider. First, due to funding constraints, this study was designed as a single-center trial with a relatively limited sample size, which may, to some extent, affect the generalizability of the findings. Moreover, the assumed between-group difference used in the sample size calculation may have been overly optimistic, resulting in insufficient statistical power to detect more subtle yet clinically meaningful effects of shallow needling. Nevertheless, our study offers a valuable foundation for understanding the efficacy of shallow needling, and we hope that future large-scale, multicenter studies will further validate and extend these findings, thereby strengthening the evidence base for this intervention. Second, all participants received baseline pharmacotherapy with eszopiclone for ethical and adherence reasons, which may introduce confounding effects when interpreting the specific contribution of shallow needling. Therefore, we incorporated a follow-up period to reduce the residual pharmacological effects and more accurately assess the long-term efficacy attributable to shallow needling therapy. Finally, given the unique characteristics of shallow needling, developing a sham intervention that is both physiologically inert and indistinguishable from the actual treatment is difficult. Any form of simulated contact or scraping may inadvertently induce physiological reactions, thereby compromising its validity as a true placebo. Therefore, we adopted multidimensional objective outcome measures and blinded the evaluators and statisticians to reduce potential bias risks. At the same time, a 4-week follow-up was conducted to reduce the short-term placebo effect and enhance the validity of the research results. We hope that future research can develop and apply innovative sham-control methods tailored to shallow needling techniques to further reduce the possible placebo bias.

To the best of our knowledge, this is the first randomized controlled trial to objectively assess the efficacy of shallow needling for the treatment of insomnia and provide new insights into its neurophysiological mechanisms. The results of this study will establish a basis for the wider use of shallow needling in clinical practice and provide fresh evidence-based support for its application in treating insomnia.

## Supplementary material

10.2196/76501 Checklist 1 SPIRIT checklist.

## Abbreviations

CBT: cognitive behavioral therapy
CRF: case report form
GABA: γ-aminobutyric acid
Glx: glutamate/glutamine
MRS: magnetic resonance spectroscopy
PI: primary insomnia
PSQI: Pittsburgh Sleep Quality Index
REM: rapid eye movement
SPIRIT: Standard Protocol Items: Recommendations for Interventional Trials
